# Visual and rapid identification of *Chlamydia trachomatis* and *Neisseria gonorrhoeae* using multiplex loop-mediated isothermal amplification and a gold nanoparticle-based lateral flow biosensor

**DOI:** 10.3389/fcimb.2023.1067554

**Published:** 2023-02-28

**Authors:** Xu Chen, Qingxue Zhou, Wei Yuan, Yuanfang Shi, Shilei Dong, Xinhua Luo

**Affiliations:** ^1^The Second Clinical College, Guizhou University of Traditional Chinese Medicine, Guiyang, Guizhou, China; ^2^Clinical Medical Laboratory of the Second Affiliated Hospital, Guizhou University of Traditional Chinese Medicine, Guiyang, Guizhou, China; ^3^Clinical Laboratory, Hangzhou Women’s Hospital, Hangzhou, Zhejiang, China; ^4^Department of Quality Control, Guizhou Provincial Center for Clinical Laboratory, Guiyang, Guizhou, China; ^5^Department of Clinical Laboratory, Zhejiang Hospital, Hangzhou, Zhejiang, China; ^6^Department of Infectious Disease, Guizhou Provincial People’s Hospital, Guiyang, Guizhou, China

**Keywords:** *Chlamydia trachomatis*, *Neisseria gonorrhoeae*, loop-mediated isothermal amplification, gold nanoparticle-based lateral flow biosensor, point-of-care testing

## Abstract

Sexually transmitted chlamydia and gonorrhea infections caused by the bacteria *Chlamydia trachomatis* and *Neisseria gonorrhoeae* remain a major public health concern worldwide, particularly in less developed nations. It is crucial to use a point of care (POC) diagnostic method that is quick, specific, sensitive, and user-friendly to treat and control these infections effectively. Here, a novel molecular diagnostic assay, combining multiplex loop-mediated isothermal amplification (mLAMP) with a visual gold nanoparticles-based lateral flow biosensor (AuNPs-LFB) was devised and used for highly specific, sensitive, rapid, visual, and easy identification of *C. trachomatis* and *N. gonorrhoeae*. Two unique independent primer pairs were successful designed against the *ompA* and *orf1* genes of *C. trachomatis* and *N. gonorrhoeae*, respectively. The optimal mLAMP-AuNPs-LFB reaction conditions were determined to be 67°C for 35 min. The detection procedure, involving crude genomic DNA extraction (~5 min), LAMP amplification (35 min), and visual results interpretation (<2 min), can be completed within 45 min. Our assay has a detection limit of 50 copies per test, and we did not observe any cross-reactivity with any other bacteria in our testing. Hence, our mLAMP-AuNPs-LFB assay can potentially be used for POC testing to detect *C. trachomatis* and *N. gonorrhoeae* in clinical settings, particularly in underdeveloped regions.

## Introduction

Sexually transmitted infections (STIs) are a major global public health concern and a cause of serious genital and reproductive morbidity and mortality, especially in underdeveloped regions (Kojima and Klausner, 2021;Tsevat et al., 2017). *Chlamydia trachomatis* and *Neisseria gonorrhoeae* are the two most common pathogens responsible for STIs, with around 130 and 87 million new cases annually worldwide, respectively, according to the World Health Organization (WHO) (World Health Organization, 2016; Rowley et al., 2019). Both infections are mainly asymptomatic, but if left untreated, they can lead to many severe complications (Foschi et al., 2020). They are strongly associated with orchitis, prostatitis, epididymitis, and urethritis in males (Wilkinson et al., 2021). Infections may result in pelvic inflammatory illness, salpingitis, ectopic pregnancy, and infertility in females (Uprety and Cardenas, 2019;Herzog et al., 2012). Fetal infection, premature delivery, stillbirth, low birth weight, and miscarriage can arise during pregnancy with infected mothers (Gaydos et al., 2019; Niles et al., 2021). Furthermore, *C. trachomatis* and *N. gonorrhoeae* are associated with a higher risk of human immunodeficiency virus infections (Tsevat et al., 2017; Goulart et al., 2020; Wilkinson et al., 2021). Therefore, developing point-of-care (POC) diagnostic instruments that are fast, sensitive, specific, and easily understandable is crucial to treat and contain these infections.

Loop-mediated isothermal amplification (LAMP) is an intriguing alternative to the polymerase chain reaction (PCR) and PCR-related technologies for POC nucleic acid testing (Augustine et al., 2020; Chaouch, 2021). Strong target gene amplification is achieved by performing nucleic acid isothermal amplification at a fixed temperature (58°C–69°C) for 30–60 min using *Bacillus stearothermophilus* (*Bst*) DNA polymerase with strand-displacement activity (Notomi et al., 2000; Shang et al., 2020). Two outside primers (F3 and B3), two interior primers (FIP and BIP), and two loop primers (LF and LB) are designed to span eight different target fragment areas to amplify target gene sequences (Varona and Anderson, 2021). The LAMP method has previously been used for the immediate, specific, sensitive, and affordable detection of pathogens such as the severe acute respiratory syndrome coronavirus 2, Zika virus, and human papillomavirus (Silva et al., 2020; Janikova et al., 2021; Vo and Story, 2021). Meanwhile, the LAMP has already been used to identify *C. trachomatis* and *N. gonorrhoeae*. Choopara et al. and Somboonna et al. combined LAMP with hydroxynaphthol blue for visual detection of *C. trachomatis* by the naked eye (Choopara et al., 2017; Somboonna and Choopara, 2019). Liu et al. combined LAMP with fluorescence dye for visual identification of *N. gonorrhoeae* by the naked eye (Liu et al., 2017). However, the results of each assay were ambiguous when the LAMP product concentration was low, and is inadequate for simultaneously detecting two target genes in a single test. Eboigbodin et al. combined LAMP with fluorescence reader for simultaneously detection of *C. trachomatis* and *N. gonorrhoeae* (Eboigbodin, 2019). However, this assay requires expensive equipments.

**Figure 1:**
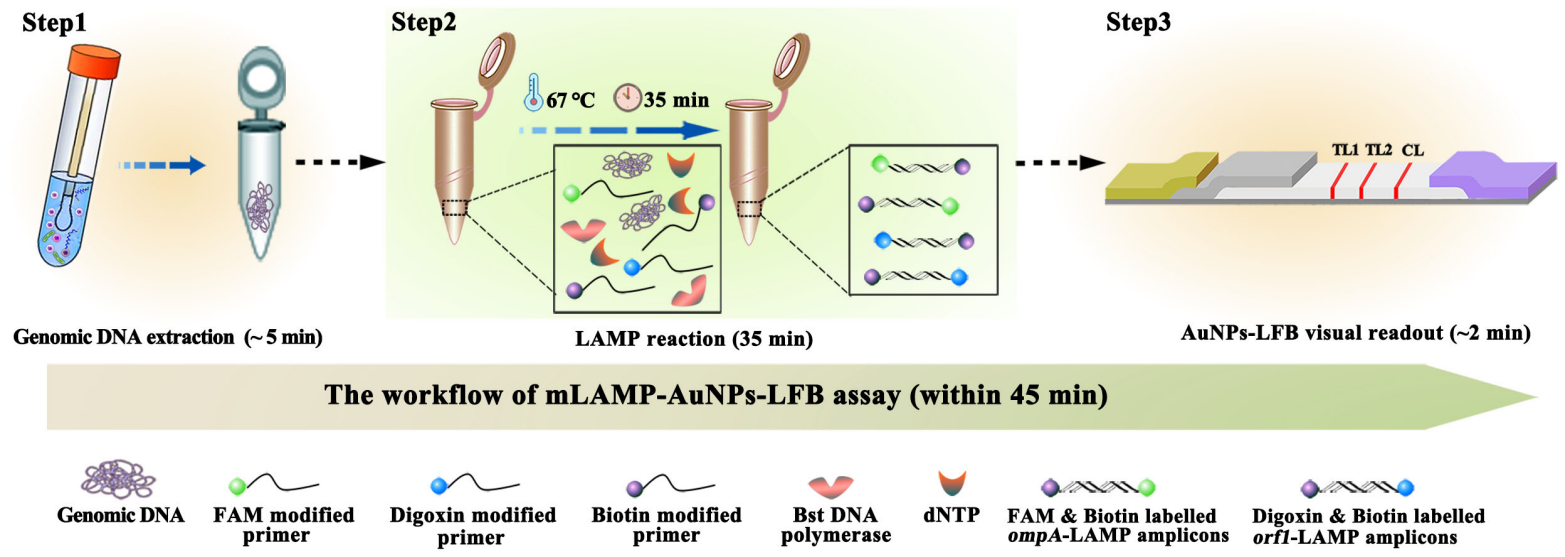
mLAMP-AuNPs-LFB assay workflow The mLAMP-AuNPs-LFB assay’s workflow includes genomic DNA extraction (5 min), mLAMP reaction (35 min), and AuNPs-LFB visual readout (<2 min) and can be completed within 45 min.

**Figure 2:**
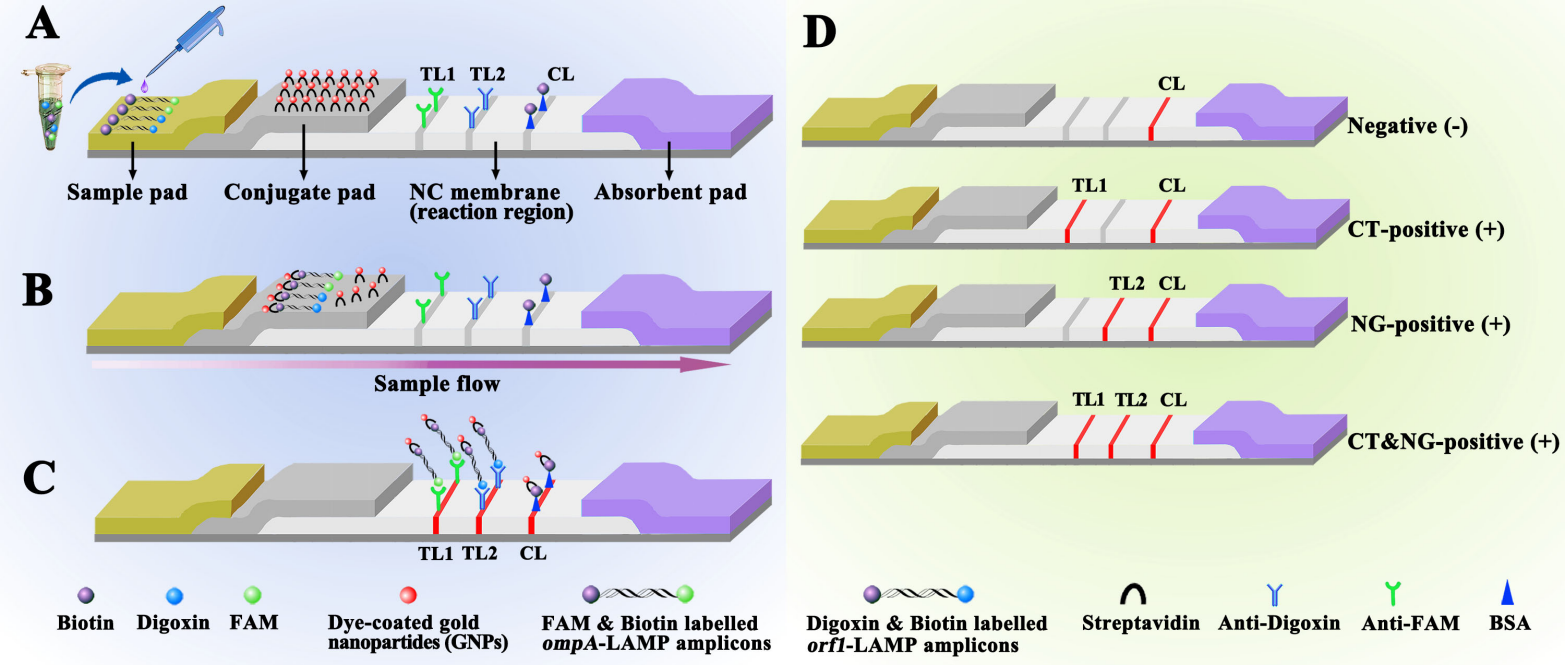
Schematic diagram showing AuNPs-LFB principles for visual mLAMP amplification product interpretation **(A)** mLAMP amplification products (0.5 μL) and running buffer (100 μL) were simultaneously added to the sample pad. **(B)** The running buffer containing the mLAMP products moves forward onto the conjugate pad and nitrocellulose membrane by capillary action. SA-AuNPss are hydrated, rapidly released, and combined with *C*. *trachomatis* or *N. gonorrhoeae* LAMP products at the conjugate pad. **(C)** FAM/biotin-labeled *C*. *trachomatis* LAMP products are captured by anti-FAM at TL1, Dig/biotin-labeled *N. gonorrhoeae* LAMP products are captured by anti-Dig at TL2, and SA-AuNPs are captured by biotin-BSA at the CL. **(D)** Interpretation of the mLAMP-AuNPs-LFB assay. *C*. *trachomatis* positive results are indicated by CL and TL1 bands on the AuNPs-LFB, *N. gonorrhoeae* positive results are indicated by CL and TL2 bands on the AuNPs-LFB, and *C*. *trachomatis* and *N. gonorrhoeae* positive results are indicated by TL1, TL2, and CL bands on the AuNPs-LFB. Negative results are indicated when only the CL band appears on the AuNPs-LFB. Key: CL, control line; TL1, test line one; TL2, test line two; CT, *C*. *trachomatis*; NG, *N. gonorrhoeae*; NC, nitrocellulose membrane.

In order to overcome these shortcomings, the paper- and gold nanoparticle (AuNPs)-based lateral flow biosensor (LFB) platform is designed and widely recognized as an ideal POC diagnostic testing method due to its high reliability, selectivity, accuracy, minimal maintenance, prolonged stability, and limit of detection (LoD) (Quesada-González and Merkoçi, 2015;Kim et al., 2019). Recent advances in using AuNPs as biosensors have enabled AuNPs-LFBs to identify various biomarkers, including proteins, nucleic acids, and even entire cells (Koczula and Gallotta, 2016; Wang et al., 2021). Based on these features, AuNPs-LFBs are already widely used in biomedicine, agriculture, food safety, and environmental sciences (Aldewachi et al., 2017).In this study, multiplex LAMP (mLAMP) was combined with an AuNPs-based LFB (mLAMP-AuNPs-LFB) to rapidly, simply, and visually identify *C. trachomatis* and *N. gonorrhoeae* by targeting their *ompA* and *orf1* genes, respectively (Edwards et al., 2014; Somboonna et al., 2018). Both genes showed no homology to other microbial genomes in BLAST searches of the GenBank database. The mLAMP-AuNPs-LFB assay principle and workflow are shown in **Figures 1**, **2**, respectively. Its feasibility was confirmed using clinical genital secretion samples from patients. This assay can be completed within 45 min without any expensive facilities. Therefore, the mLAMP-AuNPs-LFB assay appears promising for POC testing to identify *C. trachomatis* and *N. gonorrhoeae*, particularly in situations with limited diagnostic tools.

## Materials and methods

### Reagents

We obtained AuNPs-based LFB materials, including biotinylated bovine serum albumin (biotin-BSA; 4 mg/mL), a rabbit anti-fluorescein antibody (anti-FAM; 0.2 mg/mL), and a sheep anti-digoxigenin antibody (anti-Dig; 0.25 mg/mL) from Abcam Co., Ltd. (Shanghai, China), and crimson red dye streptavidin-coated AuNPs (SA-AuNPs; 40 ± 5 nm, 10 mg/mL, 100 mM borate [pH 8.5] with 0.1% BSA, 0.05% Tween 20, and 10 mM ethylenediaminetetraacetic acid) from Bangs Laboratories Inc. (Fishers, IN, USA). Four LFB components (sample, conjugate, absorption pads, and nitrocellulose membranes) were manufactured and laminated on plastic adhesive backing by HuiDeXin Biotech. Co., Ltd. (Tianjin, China) according to our design scheme (**Figure 2**). Nucleic acid releasers were obtained from BaiAoLaiBo Technique Ltd. (Beijing, China). Universal isothermal amplification kits and the colorimetric indicator, malachite green (MG), were obtained from HuiDeXin Biotech. Co., Ltd. (Tianjin, China). Commercial PCR diagnostic kits for *C. trachomatis* and *N. gonorrhoeae* were obtained from DaAn Gene Co., Ltd. (Guangzhou, China).

### Preparing target DNA and clinical samples

Full-length *ompA* sequences from 14 C*. trachomatis* serological variants (A-K and L1-L3; GenBank accession numbers JX548318.1, JX559518.1, JX559519.1, KP164991.1, JX559522.1, JX564244.1, JX564245.1, JX564246.1, JX564247.1, JX648604.1, JX564248.1, JX569832.1, KP120855.1, and JX569834.1) and the *N. gonorrhoeae orf1* gene (M84113.1) were synthetically produced and cloned into the pUC57 vector. The basal concentration of all plasmids was 1×10^8^ copies. The synthesized plasmids were used as positive controls.

Participants at the Hangzhou Women’s Hospital provided 146 vaginal discharge samples for examination between August 2021 and March 2022 that were believed to include STIs caused by *C. trachomatis* and/or *N. gonorrhoeae*. Nucleic acid-releasing agents were used to extract crude genomic DNA according to the manufacturer’s guidelines. Briefly, a sample was incubated with 100 μl of a nucleic acid releasing agent for 5 min. The resulting supernatant was used as a template for LAMP amplification. The quantities of genomic DNA were calculated using the 260/280 nm absorbance ratio using a Nano-Drop ND 2000 instrument (Thermo Fisher Scientific; Waltham, MA, USA). Other microorganisms investigated in this study are listed in **Table 1**.

**Table 1:** Microbial strains used in this study.

No.	Pathogen	Source of strains^a^	No. of strains	mLAMP-AuNPs-LFB result^b^	
				*C. trachomatis*	*N. gonorrhoeae*
1	*C. trachomatis* serovar A-K, L1-L3 *ompA*-plasmids	Tsingke Biotech (Beijing, China)	14	P	N
2	*C. trachomatis* (clinical samples)	Hangzhou Women’s Hospital	6	P	N
3	*N. gonorrhoeae orf1*-plasmids	Tsingke Biotech (Beijing, China)	1	N	P
4	*N. gonorrhoeae* (reference strain)	ATCC 49926	1	N	P
5	*N. gonorrhoeae* (clinical samples)	Hangzhou Women’s Hospital	6	N	P
6	*C. trachomatis and N. gonorrhoeae* (clinical samples)	Hangzhou Women’s Hospital	6	P	P
7	*Neisseria meningitides*	Hangzhou Women’s Hospital	1	N	N
8	*Ureaplasma urealyticum*	Hangzhou Women’s Hospital	1	N	N
9	*Escherichia coli*	2^nd^ GZUTCM	1	N	N
10	*Staphylococcus aureus*	2^nd^ GZUTCM	1	N	N
11	Human papilloma virus	GZCCL	1	N	N
12	*Mycoplasma pneumoniae*	Zhejiang Hospital	1	N	N
13	*Haemophilus influenza*	ATCC49247	1	N	N
14	*Streptococcus pyogenes*	2^nd^ GZUTCM	1	N	N
15	Human enterovirus EV71	GZCCL	1	N	N
16	Coxsackie virus CAV16	GZCCL	1	N	N
17	*Klebsiella pneumoniae*	Zhejiang Hospital	1	N	N
18	*Pseudomonas aeruginosa*	2^nd^ GZUTCM	1	N	N
19	*Candida glabrata*	2^nd^ GZUTCM	1	N	N
20	*Cryptococcus neoformans*	ATCC13690	1	N	N
21	*Listeria monocytogenes*	Zhejiang Hospital	1	N	N

^a^ATCC, American Type Culture Collection; 2^nd^ GZUTCM, the Second Affiliated Hospital, Guizhou University of Traditional Chinese Medicine; GZCCL, Guizhou Provincial Center for Clinical Laboratory. ^b^P, Positive; N, Negative.

### AuNPs-based biosensor construction

**Figure 2** shows that the AuNPs-LFB, with dimensions of 60 mm × 4 mm, is divided into four sections: the sample pad, the conjugate pad, the detection region (nitrocellulose membrane), and the absorption pad. SA-AuNPs containing crimson red dye are deposited on top of the conjugate pad. Rabbit anti-FAM (0.2 mg/mL), sheep anti-Dig (0.25 mg/mL), and biotin-BSA (4 mg/mL) are immobilized onto the nitrocellulose membrane of test 1 (TL1) and 2 (TL2) and control (CL) lines, respectively, which were separated by 5 mm. Finally, the four separate parts are bonded together on a plastic card with an adhesive backing. The AuNPs-LFB was kept dry and at room temperature until used. The workflow of AuNPs-LFB assay as following, LAMP products and running buffer were added to the AuNPs-LFB sample pad concurrently. A capillary action carries the LAMP product-containing flowing buffer along the biosensor, rehydrating the SA-AuNPs immobilized in crimson red dye. Biotin-BSA was used to capture SA-AuNPs at the CL. Anti-FAM was used to capture FAM/biotin-labeled *C. trachomatis*-LAMP products at TL1. Anti-Dig was used to capture Dig/biotin-labeled *N. gonorrhoeae*-LAMP amplicons at the TL2. The purpose of control line (CL) is to monitor whether the AuNPs-LFB test is valid. The CL band must be shown in each test. When the CL brand is not appeared, indicating the test is invalid.

### LAMP primer design

Two LAMP primer sets were created based on the *ompA* and *orf1* target genes to detect *C. trachomatis* and *N. gonorrhoeae*, respectively. The *ompA* genes from 14 different *C. trachomatis* serological variations (serovars A, B, C, D, E, F, G, H, I, J, K, L1, L2, and L3) were aligned using the MEGA-X software (https://www.megasoftware.net/) (**Supplementary Figure S1**). Conserved sequences were used for *C. trachomatis* LAMP primer design with the Primer Explorer v.5 (http://primerexplorer.jp/e/) and Primer Premier v.5.0 software. Primer pair specificity was confirmed using the BLAST analysis tool. **Table 2** lists the primer sequences and alterations, and **Supplementary Figure S1** shows the primer locations. All primers were produced and purified *via* high-performance liquid chromatography by The TsingKe Biotechnology Company (Beijing, China).

**Table 2:** The mLAMP-AuNPs-LFB primers used in this study.

Primer name	Sequence and modifications	Length	Gene
F3	5′-AGT(A/G)TTTGCCGCTTTGAGTTCT-3′	22 nt	*ompA*
B3	5′-AAAC(A/G)CGGTCGAAAACAAAGTC-3′	22 nt	
FIP*	5′-FAM-A(C/T)AGAATTCCGTCGATCATAAGGCTTTCCTCCTTGCAAGCTCTG-3′	44 mer	
BIP	5′-GGAAGGTTT(C/T)GG(C/T)GGAGATCC(A/T)CC(A/G)TAGTAACC(A/C)A(C/T)(A/G)CGCATG-3′	43 mer	
LF*	5′-Biotin-TCAGCAGGATTCCCCAC-3′	17 nt	
LB	5′-GC(A/G)CCACTTGGTGTGAC-3′	17 nt	
F3	5′-CGGTCAAAACCTGTTCGCA-3′	19 nt	o*rf1*
B3	5′-AGACGGGATACGGATGGAAG-3′	20 nt	
FIP*	5′-Dig-ATCGCGTTCAGGGCTTTGGCGTCCGATACCGCGCTCTA-3′	38 mer	
BIP	5′-ACCAACTCCTACAAACGCCTCGTTTGGCGGAATAGGCCAATT-3′	42 mer	
LF*	5′-Biotin-TTGATGATGCCGCCGATG-3′	18 nt	
LB	5′-CGCACTTTGAAGCACCGA-3′	18 nt	

ompA-FIP*, 5′-labeled with FAM, ompA-LF*,5′-labeled with biotin, orf1-FIP*, 5′-labeled with Dig, orf1-LF*,5′-labeled with biotin, when used for the AuNP-LFB assay.

FAM, 6-carboxy-fluorescein; Dig digoxigenin; nt, nucleotide; mer, monomeric unit.

### LAMP reactions and detection

The single LAMP reactions for *C. trachomatis* or *N. gonorrhoeae* were performed in 25 μL reaction volumes. Each reaction contained 1 μL of each standard plasmid template (5 μL of the clinical sample template); 0.8 μM of F3, B3, LF, and LB; 1.6 μM of FIP and BIP; 1 μL of *Bst* 2.0 DNA polymerase (8 U); 12.5 μL of 2× reaction buffer (2 M betaine, 16 mM magnesium sulfate, 40 mM potassium chloride, 20 mM ammonium sulfate, 40 mM Tris-hydrochloride [pH 8.8], and 0.2% Tween-20); 1 μL of the colorimetric indicator (MG); supplemented with double-distilled water (DW) to 25 μL.

The one-step mLAMP reaction was performed in a 25 μl reaction volume containing 1 μL of each standard plasmid template (5 μL of the clinical sample template); 0.3 μM of *ompA*-F3, *ompA*-B3, *ompA*-LF, and *ompA*-LB; 0.6 μM of *ompA*-FIP and *ompA*-BIP; 0.5 μM of *orf1*-F3, *orf1*-B3, *orf1*-LF, and *orf1*-LB; 1 μM each o*rf1*-FIP, and *orf1*-BIP; 12.5 μL of 2×reaction buffer; 1 μL of *Bst* 2.0 DNA polymerase (8 U); 1 μL of the colorimetric indicator (MG); supplemented with DW to 25 μl in DW water. The reaction was performed in a heat-block or water bath at a constant temperature (conditions were optimized as outlined below).

Real-time turbidity LA-500 (Lumiprobe; Tokyo, Japan), visual detection reagents (MG), and the AuNPs-LFB were used to track the LAMP amplification products. A turbidity value of >0.1 indicated a successful outcome. Reaction mixtures that turned bright green indicated a successful MG visual detection; a colorless reaction indicated a failed detection. CL and TL1 simultaneously appearing on the AuNPs-LFB indicated a positive *C. trachomatis*-LAMP result. CL and TL2 simultaneously appearing on the AuNPs-LFB indicated a positive *N. gonorrhoeae*-LAMP result. CL only appearing on the AuNPs-LFB indicated a negative result.

### Assay condition optimization

The optimal mLAMP-AuNPs-LFB reaction temperature was determined by incubating the *C. trachomatis*-LAMP and *N. gonorrhoeae*-LAMP reactions at 62°C to 69°C (in 1°C increments) and assessing their amplification results using real-time turbidity. Then, the incubation time was varied from 15 to 45 min (in 10 min increments) at the optimal reaction temperature with the amplification results detected by AuNPs-LFB. Each assay was performed in triplicate.

### mLAMP-AuNPs-LFB assay sensitivity

Two conventional plasmids, the *ompA* and *orf1* plasmids, were produced and then serially diluted by a factor of ten to provide copy numbers ranging from 5.0×10^4^ to 5.0×10^-1^. LAMP reactions were performed under optimum conditions, and the data were examined using MG and AuNPs-LFB.

### mLAMP-AuNPs-LFB assay specificity

Synthetic nucleic acid sequences and bacterial, viral, and fungal nucleic acid templates were detected at ≥1.0×10^4^ copies to assess the mLAMP-AuNPs-LFB assay’s specificity (**Table 1**). All assays were performed at least thrice, with DW serving as the negative control.

### Feasibility of the mLAMP-AuNPs-LFB for clinical samples

Genital secretion samples were obtained from 146 women at the Hangzhou Women’s Hospital who were believed to have STIs caused by *C. trachomatis* and/or *N. gonorrhoeae*. Genomic DNA templates were obtained rapidly using the Nucleic Acid Releasers Kit (BaiAoLaiBo Technique Ltd.) according to the manufacturer’s guidelines. We compared the mLAMP-AuNPs-LFB assay to commercially available real-time TaqMan PCR Kits for *C. trachomatis* and *N. gonorrhoeae* (DaAn Gene Co., Ltd.; Guangzhou, China) on an Applied Biosystems™ 7500 Real-Time PCR System (Life Technologies; Singapore). The basis for determining the copy number of the samples according to the corresponding standard curve. *C. trachomatis* and *N. gonorrhoeae* concentrations of >500 copies were considered positive based on the manufacturer’s recommendations. The Human Ethics Committee of Hangzhou Women’s Hospital approved the lawful collection and analysis of these genomic DNA templates (approval no. [2021]-K[2]-8). The identification investigations were performed at biosafety level 2 based on the WHO Biosafety Manual (3^rd^ Edition). The mLAMP-AuNPs-LFB data were compared with quantitative real-time PCR (qPCR) data.

## Results

### mLAMP-AuNPs-LFB assay system overview

A representative schematic and workflow of the mLAMP-AuNPs-LFB assay are shown in **Figures 1**, **2**, respectively. Briefly, genomic DNA templates were rapidly extracted using nucleic acid releasers (**Figure 1**, step 1). The *ompA*-FIP* and *ompA*-LF* primers were 5’-labeled with fluorescein (FAM) and biotin, and *orf1*-FIP* and *orf1*-LF* with digoxigenin (Dig) and biotin. The *ompA*-LAMP products were labeled with FAM and biotin and the *orf1*-LAMP products with Dig and biotin after a 35 min reaction at 67°C (**Figure 1**, step 2). Finally, the mLAMP products were visually monitored with an AuNPs-LFB within 2 min (**Figure 1**, step 3).

LAMP products (0.5 µL) and running buffer (100 µL; 100 mM phosphate-buffered saline with 1% Tween 20 [pH 7.4]) were added to the AuNPs-LFB sample pad concurrently (**Figure 2A**). A capillary action carries the LAMP product-containing flowing buffer along the biosensor, rehydrating the SA-AuNPs immobilized in crimson red dye (**Figure 2B**). Biotin-BSA was used to capture SA-AuNPs at the CL. Anti-FAM was used to capture FAM/biotin-labeled *ompA*-LAMP products at TL1. Anti-Dig was used to capture Dig/biotin-labeled *orf1*-LAMP amplicons at the TL2. The result is negative when only biotin-BSA captures the SA-AuNPs at the CL (**Figure 2C**). The interpretation of the mLAMP-AuNPs-LFB assay is outlined in **Figure 2D**.

### mLAMP-AuNPs-LFB assay confirmation

**Figure 3:**
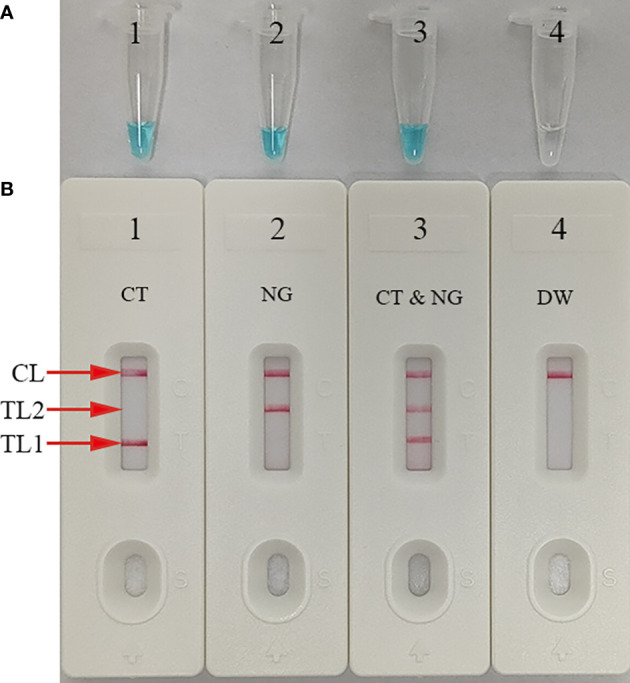
mLAMP product confirmation and verification The mLAMP products were simultaneously identified using **(A)** malachite green and **(B)** the AuNPs-LFB. Tube/AuNPs-LFB 1 shows a positive *C*. *trachomatis* result. Tube/AuNPs-LFB 2 shows a positive *N. gonorrhoeae* result. Tube/AuNPs-LFB 3 shows a positive *C*. *trachomatis* and *N. gonorrhoeae* result. Tube/AuNPs-LFB 4 shows negative control (DW). Key: CL, control line; TL1, test line one; TL2, test line two; CT, *C*. *trachomatis*; NG, *N. gonorrhoeae*.

The *ompA*-, *orf1*-, or mLAMP-amplification mixes were incubated at a fixed temperature (65°C) for 1 h to confirm the reaction system with the two LAMP primer sets. Then, a visual detection reagent (MG) and the AuNPs-LFB were used to assess the *ompA*/*orf1* and mLAMP amplification results, respectively. DW was used as the negative control. While positive *ompA*-, *orf1*-, and mLAMP reactions changed from colorlessness to bright green, the negative control remained colorless (**Figure 3A**). The co-appearance of crimson-red TL1 and CL bands with AuNPs-LFB detection indicated a positive *ompA*-LAMP result. The co-appearance of crimson-red TL2 and CL bands indicated a positive *orf1*-LAMP result. A positive mLAMP result was indicated by the simultaneous appearance of TL1, TL2, and CL bands in the AuNPs-LFB. In contrast, the negative control showed only a crimson red CL band (**Figure 3B**). In conclusion, our findings supported using both LAMP primer sets in the mLAMP reaction.

### *ompA*- and *orf1*-LAMP assay temperature optimization

**Figure 4:**
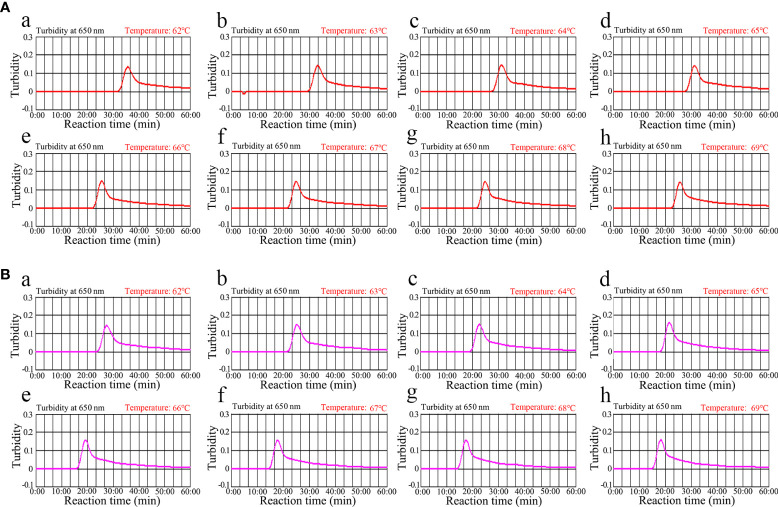
Temperature optimization for the (*C*) *trachomatis* and *N. gonorrhoeae* LAMP amplification LAMP amplifications for **(A)** *C*. *trachomatis* and **(B)** *N. gonorrhoeae* were monitored using real-time turbidity, and their corresponding amplicon curves are shown as graphs. A turbidity >0.1 indicated a positive result. Eight kinetic graphs were obtained at different temperatures (62°C–69°C in 1°C increments) with 1×10^4^ target gene copies. Graphs **e** (66°C) to **h** (69°C) in **(A)** showed robust amplification. Graphs from **f** (67°C) to **h** (69°C) in **(B)** showed robust amplification.

Optimizing the incubation temperature for LAMP reactions is essential. Therefore, we used a standard plasmid copy number (1.0×10^4^) to evaluate temperatures from 62°C to 69°C in 1°C increments. The LAMP amplification results were tracked in real-time using turbidity measurements. The results indicated that robust *ompA*-LAMP amplification occurred at 66°C to 69°C and *orf1*-LAMP amplification at 67°C to 69°C (**Figure 4**). Therefore, 67°C was the most suitable amplification temperature for our assay.

### mLAMP-AuNPs-LFB assay sensitivity

**Figure 5:**
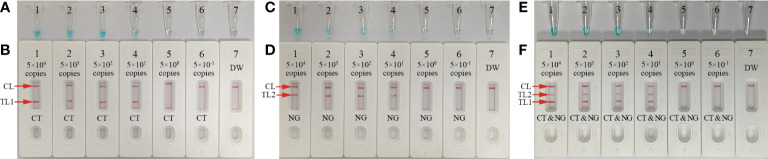
Sensitivity analysis of the mLAMP-AuNPs-LFB assay with serial nucleic acid template dilutions Serial dilutions (5.0×10^4^, 5.0×10^3^, 5.0×10^2^, 5.0×10^1^, 5.0×10^0^, and 5.0×10^-1^ copies) of *C. trachomatis ompA* and *N. gonorrhoeae orf1* plasmids were used as templates, and DW was used as the negative control. Results were simultaneously analyzed by visual reagent malachite green and the AuNPs-LFB. **(A, B)**: A sensitivity analysis of the *C. trachomatis* LAMP assay indicated its LoD was 50 copies per reaction. **(C, D)**: A sensitivity analysis of the *N. gonorrhoeae* LAMP assay indicated its LoD was 50 copies per reaction. **(E, F)**: A sensitivity analysis of the mLAMP assay for *ompA* and *orf1* indicated its LoD was 50 copies of the nucleic acid template per reaction. Key: CL, control line; TL1, test line one; TL2, test line two; CT, *C. trachomatis*; NG, *N. gonorrhoeae*; MG, malachite green.

Serial dilutions of nucleic acid templates (*ompA*- and *orf1*-plasmid constructs) with 5.0×10^4^ to 5.0×10^-1^ copies were used to determine the LoD of our assay. mLAMP reactions were performed as described above, and the results were visualized using MG and the AuNPs-LFB. The sensitivity of our assay was 50 copies per test for both the *ompA* (**Figures 5A, B**) and *orf1* (**Figures 5C, D**) plasmids. The two target genes were simultaneously detected and identified in a single reaction (**Figures 5E, F**). The mLAMP results using the AuNPs-LFB were consistent with the visual detection reagent (MG; **Figure 5**). However, it should be noted that the visual detection method (MG) cannot discriminate target genes in a multiplex assay. The sensitivity of mLAMP-AuNPs-LFB was consistent with single *ompA* and *orf1* mLAMP-AuNPs-LFB assays (**Figure 5**).

### mLAMP-AuNPs-LFB assay reaction time optimization

**Figure 6:**
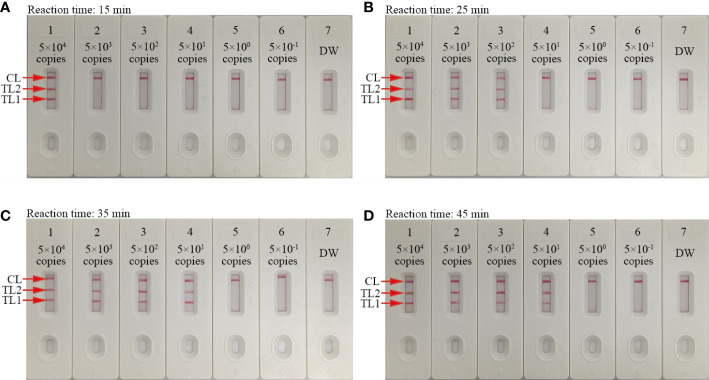
Amplification time optimization for the mLAMP-AuNPs-LFB assay Four LAMP reaction times were evaluated at 67°C: **(A)** 15 min, **(B)** 25 min, **(C)** 35 min, and **(D)** 45 min. AuNPs-LFBs 1–7 represent nucleic acid template levels 5.0×10^4^, 5.0×10^3^, 5.0×10^2^, 5.0×10^1^, 5.0×10^0^, and 5.0×10^-1^ copies and negative control (DW), respectively. Results were analyzed using AuNPs-LFBs. The optimal LoD occurred with a 35 min reaction time **(C)**. Key: CL, control line; TL1, test line one; TL2, test line two.

Four assay reaction times (15, 25, 35, and 45 min) were evaluated at 67°C to determine the optimal reaction time for the LAMP amplification stage of the protocol. The AuNPs-LFB was used to determine the outcomes. A 35 min LAMP reaction was found to be optimal at the genomic DNA template LoD (50 copies; **Figure 6**). Therefore, our assay’s entire detection process can be completed within 45 min: rapid genomic DNA preparation (5 min), mLAMP amplification (35 min), and visual outcome (<2 min).

### mLAMP-AuNPs-LFB assay specificity

**Figure 7:**
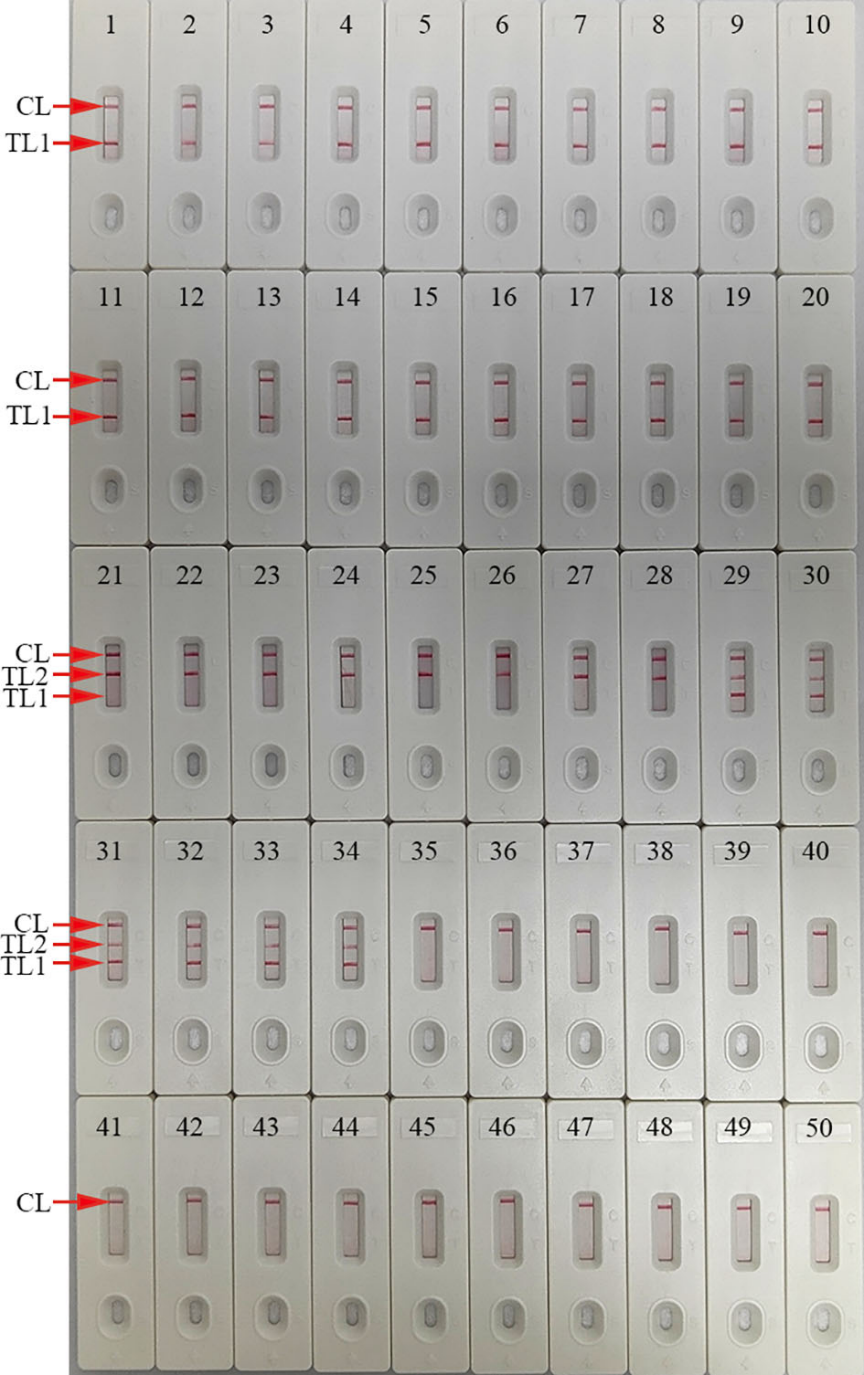
mLAMP-AuNPs-LFB assay specificity with different strains Assay specificity was evaluated using different nucleic acid templates. Amplification products were tested using AuNPs-LFBs: 1–14, *C. trachomatis* serovars A, B, C, D, E, F, G, H, I, J, K, L1, L2, and L3 *ompA* plasmids; 15–20, *C. trachomatis* clinical samples; 21, *N. gonorrhoeae orf1* plasmids; 22, *N. gonorrhoeae* reference strain ATCC 49926; 23–28, *N. gonorrhoeae* clinical samples; 29–34, *C. trachomatis* and *N. gonorrhoeae* clinical samples; 35, *Neisseria meningitides*; 36, *Ureaplasma urealyticum*; 37, *Escherichia coli*; 38, *Staphylococcus aureus*; 39, human papillomavirus; 40, *Mycoplasma pneumonia*; 41, *Haemophilus influenza*; 42, *Streptococcus pyogenes*; 43, human enterovirus EV71; 44, Coxsackie virus CAV16; 45, *Klebsiella pneumoniae*; 46, *Pseudomonas aeruginosa*;47, *Candida glabrata*; 48, *Cryptococcus neoformans*; 49, *Listeria monocytogenes*; 50, negative control (DW). Key: CL, control line; TL, test line.

Our assay’s specificity was evaluated using *C. trachomatis ompA* plasmids (serovars A, B, C, D, E, F, G, H, I, G, K, L1, L2, and L3), an *N. gonorrhoeae orf1* plasmid, positive *C. trachomatis* and *N. gonorrhoeae* clinical samples (confirmed by qPCR), and 15 other pathogens (**Table 1**). Both *C. trachomatis* and *N. gonorrhoeae* strains’ genomic DNA extractions were successful. Positive *C. trachomatis* and *N. gonorrhoeae* results were indicated by the presence of three red bands (TL1, TL2, and CL) on the AuNPs-LFB. *C. trachomatis* isolates caused TL1 and CL bands on the AuNPs-LFB, indicating success. *N. gonorrhoeae* strains caused TL2 and CL bands on the AuNPs-LFB, indicating success. All other pathogens and control groups examined returned negative results (**Figure 7**). Therefore, our assay could distinguish between *C. trachomatis* and *N. gonorrhoeae* strains.

### Evaluation of mLAMP-AuNPs-LFB assay with clinical samples

We further evaluated the clinical feasibility of our mLAMP-AuNPs-LFB assay for accurately identifying *C. trachomatis* and *N. gonorrhoeae* with 146 suspected *C. trachomatis* and/or *N. gonorrhoeae* genital secretion samples examined using qPCR and our assay. Forty-eight of the 146 samples were qPCR-confirmed (>500 copies) as *C. trachomatis*-positive, 29 as *N. gonorrhoeae*-positive, and six as *C. trachomatis* and *N. gonorrhoeae* co-infections. All *C. trachomatis* and *N. gonorrhoeae* positive samples were confirmed by our mLAMP-AuNPs-LFB assay. In addition, three *C. trachomatis*-negative and one *N. gonorrhoeae*-negative samples (genomic DNA concentrations of 100–500 copies) were positive with our mLAMP-AuNPs-LFB assay (**Table 3** and **Supplementary Table S1**). Our assay’s positive results with these four discordant samples were confirmed by PCR amplification and DNA sequencing (**Supplementary Table S1**). Therefore, these data indicate that our assay can function as an advanced clinical diagnostic tool for *C. trachomatis* and *N. gonorrhoeae*.

**Table 3:** Comparing *C. trachomatis* and *N. gonorrhoeae* levels in clinical samples using our mLAMP-AuNPs-LFB assay and qPCR method.

Detection method	Clinical samples (n=146)			
	CT positive	NG positive	CT and NG positive	Negative
qPCR	48 (> 500 copies)	29 (> 500 copies)	6 (> 500 copies)	63 (59, undetected; 3, the concentrations of *C. trachomatis* range from 100 to 500 copies; 1, the concentrations of *N. gonorrhoeae* range feom 100 to 500 copies
mLAMP-AuNPs-LFB	51	30	6	59

CT, C. trachomatis; NG, N. gonorrhoeae.

## Discussion

This study successfully developed and tested a novel mLAMP-AuNPs-LFB POC testing platform to identify *C. trachomatis* and *N. gonorrhoeae*. It uses mLAMP for *ompA* and *orf1* amplification, followed by a visual AuNPs-LFB readout. It was used to test genital secretion samples from individuals with suspected STIs caused by *C. trachomatis* and/or *N. gonorrhoeae*, and its results were compared to a commercial qPCR assay.

Various laboratory diagnostic approaches have recently been used to test chlamydia and gonorrhea and have changed considerably (Uprety and Cardenas, 2019;Adamson et al., 2020; Adamson and Klausner, 2022). Cultivation was considered the initial gold standard for *C. trachomatis* and *N. gonorrhoeae* identification (Centers for Disease Control and Prevention, 2014). However, both pathogens are fastidious and require stringent transport and culture conditions (Centers for Disease Control and Prevention, 2014). Furthermore, culture methods for *C. trachomatis* and *N. gonorrhoeae* isolation are difficult to standardize, are labor-intensive, have long turnaround times (48–72 h), require experienced analysts, and have relatively low sensitivity (Eboigbodin, 2019; Zhai et al., 2021). Serologic tests are not recommended because they lack precision for identifying active infections (Centers for Disease Control and Prevention, 2014). Nucleic acid amplification tests (NAATs) are currently recommended for diagnosing *C. trachomatis* and *N. gonorrhoeae* infections due to their high specificity and sensitivity and ease of sample transport and storage (Adamson et al., 2020; Inoue et al., 2021). Inoue et al. used multiplex PCR to simultaneously detect *N. gonorrhoeae* and *C. trachomatis* and identified 100 copies within 30 min (Inoue et al., 2021). While NAATs have improved STI diagnostic ability, they require expensive thermal cyclers and specific laboratory infrastructure, limiting accessibility in low- and middle-income countries. Our mLAMP-AuNPs-LFB POC assay only needs simple facilities and basic equipment, such as a water bath, heating block, or even a thermos cup, that can maintain reactions at 67°C for 30 min to perform the LAMP amplification step. Then, the outcomes can be visually interpreted using the AuNPs-LFB. The *Bst* DNA polymerase used in the LAMP reaction has fewer inhibitors than the *Taq* DNA polymerase used in traditional PCR. Therefore, crude genomic DNA is sufficient for LAMP amplification (Somboonna et al., 2018). Consequently, our mLAMP-AuNPs-LFB assay, which comprises crude genomic DNA extraction (~5 min), LAMP amplification (35 min), and visual result interpretation (<2 min), can be completed within 45 min.

Isothermal amplification technologies, such as recombinase polymerase (RPA) and cross-priming (CPA) amplification, have also been used to identify *C. trachomatis* and *N. gonorrhoeae*. Zhai et al. used multiplex RPA to detect *C. trachomatis* and *N. gonorrhoeae* and identified 200 copies per test within 25 min (Zhai et al., 2021). Yu et al. used multiplex CPA and detected 45 and 65 copies per test for *C. trachomatis* and *N. gonorrhoeae*, respectively, within 1.5 h (Yu et al., 2016).

This study used LAMP to pre-amplify the target genes, rapidly and robustly amplifying DNA amplicons at a constant temperature and providing 100-fold greater detection capability than traditional PCR (Notomi et al., 2000; Avendano and Patarroyo, 2020). Extremely specific amplicons are generated in the LAMP amplification system using the *Bst* DNA polymerase with four or six primer sets that span 6 or 8 unique sections of the target sequence (Notomi et al., 2000). The primer set includes forward and reverse outer (F3 and B3) and inner (FIP and BIP) primers; the four primers are usually sufficient for amplification. The reaction’s efficiency and specificity can be improved by incorporating two extra loop primers (LF and LB) into the LAMP reaction (McGinnis et al., 2021). We have previously used LAMP to detect *C. trachomatis* and *N. gonorrhoeae*, respectively, with an LoD as low as 50 copies per test (Chen et al., 2021; Chen et al., 2022). However, it was inadequate for simultaneously detecting these two important pathogens in a single test.

This study used the mLAMP method to simultaneously pre-amplify two target genes for *C. trachomatis* and *N. gonorrhoeae*. LAMP primer sets specific to the *C. trachomatis ompA* gene and *N. gonorrhoeae orf1* gene were successfully designed. *C. trachomatis* LAMP primers were designed to target eight areas of the *ompA* gene based on 14 serological *C. trachomatis* variations (serovars A-K and L1-L3). The *N. gonorrhoeae* LAMP primers were designed to target eight areas within the *orf1* gene.

The specificity of the mLAMP-AuNPs-LFB assay was confirmed with several *C. trachomatis* serological variants (A-K and L1-L3), *N. gonorrhoeae* strains, and 15 other pathogens. Our assay showed 100% specificity to both *C. trachomatis’ ompA* gene and *N. gonorrhoeae’s orf1* gene (**Table 1** and **Figure 7**). Furthermore, its LoD was as low as 50 copies (**Figure 6**). We also examined 146 genital secretion samples for *C. trachomatis* and *N. gonorrhoeae* using qPCR and our mLAMP-AuNPs-LFB assay to assess its clinical practicality. The data indicated that our assay had high specificity and sensitivity in detecting genital secretion samples (**Tables 3**, **S1**). Nevertheless, a second trial should examine clinical samples with significantly lower copy numbers using our assay to better reflect clinical applications.

An AuNPs-LFB was used in our assay for visual and rapid readout of mLAMP results. This paper-based platform is extremely applicable to POC testing due to its easy operation, sensitivity, specificity, low cost, and visual interpretation (Cui and Zhou, 2020;Huang et al., 2019). The biosensor had four parts: the sample, conjugate, adsorbent pads, and the nitrocellulose membrane. The cellulose sample pad was an excellent medium for delivering the examined mLAMP products to the biosensor elements. The conjugate pad contained crimson red dyed SA-AuNPs. Nitrocellulose membranes immobilized target reactive molecules, such as anti-FAM, anti-Dig, and BSA-biotin, at the TL1, TL2, and CL positions, respectively. All three antigens (CL, TL1, and TL2) appeared simultaneously on the AuNPs-LFB for *C. trachomatis*- and *N. gonorrhoeae*-positive samples. CL and TL1 appeared simultaneously on the AuNPs-LFB for *C. trachomatis*-positive samples. CL and TL2 appeared simultaneously on the AuNPs-LFB for *N. gonorrhoeae*-positive samples. Only CL appeared on the AuNPs-LFB for *C. trachomatis*- and *N. gonorrhoeae*-negative samples. The adsorbent pad acts as a bibulous paper to direct the LAMP product flow from the sample pad to the reaction zone. It is located at the tip of the AuNPs-LFB (nitrocellulose membrane). A turbidimeter and visual reagent (MG) were also used to analyze mLAMP results. However, each requires specific devices or reagents and is inadequate for simultaneously detecting two target genes in a single test. Our AuNPs-LFB assay was also cheaper (~US$2.0/test). Therefore, the total cost for each test, including genomic DNA extraction (~US$0.50), LAMP reactions (~US$3.00), and AuNPs-LFB readout (~US$2.00), was approximately US$5.50.

Nevertheless, our AuNPs-LFB assay has some weaknesses. First, the result obtained with the naked eye is qualitative but not quantitative. Quantitative measurements with the mLAMP-AuNPs-LFB assay require further study. Second, the mLAMP reaction tube must be opened to be read by the AuNPs-LFB, increasing the risk of carry-over contamination. However, spraying 70% ethanol and 10%–15% sodium hypochlorite solution soon after completing each AuNPs-LFB assay is an effective way to avoid nucleic acid contamination. In our laboratory, cross-contamination appeared to have been effectively controlled since we encountered no false-positive results. In order to better fit the clinical application, it is necessary to improve the mLAMP-AuNPs-LFB assay system, and design a device for avoiding the opening tube procedure to reduce aerosol pollution.

## Conclusions

This study combined mLAMP isothermal amplification with a visual AuNPs-LFB interpretation to create a novel mLAMP-AuNPs-LFB assay for the highly specific, sensitive, and rapid identification of *C. trachomatis* and *N. gonorrhoeae* in clinical settings. Our assay had a 50-copy LoD and showed no cross-reactivity with other pathogens. The entire test process can be completed within 45 min and does not require sophisticated equipment. Therefore, it meets the WHO-recommended assured POC testing requirements: low cost, sensitive, specific, user friendly, robust, equipment free, and attainable.

## Data availability statement

The original contributions presented in the study are included in the article/**Supplementary Material**. Further inquiries can be directed to the corresponding authors.

## Ethics statement

This study was approved by the Human Ethics Committee of Hangzhou Women’s Hospital (Approval No. [2021]-K (2)-8).

## Author contributions

XC, SD and XL were involved in study conceptualization, supervision, and project administration. QZ, WY and YS performed experiments and data curation. XC and QZ involved in study funding acquisition and methodology. XC, QZ, WY, YS and SD collected clinical samples. QZ, WY and YS were involved in validation studies and visualization. XC was involved in writing–original draft. SD and XL were involved in writing–review and editing. All authors contributed to the article and approved the submitted version.

## Supplementary material

Supplementary Figure 1 Nucleotide sequences and location of the *C. trachomatis-ompA* and *N. gonorrhoeae*-*orf1* genes used to designed the mLAMP primers.

Supplementary Table 1 Comparison of mLAMP-AuNPs-LFB and qPCR methods for assessment of *C. trachomatis and N. gonorrhoeae* in clinical samples.
