# Supplementary material for: Visual and rapid identification of Chlamydia trachomatis and Neisseria gonorrhoeae using multiplex loop-mediated isothermal amplification and a gold nanoparticle-based lateral flow biosensor
Source: Front Cell Infect Microbiol. 2023 Feb 28;13:1067554. doi: 10.3389/fcimb.2023.1067554 (PMC10011439; doi:10.3389/fcimb.2023.1067554)
Supplement: Supplementary Figure 1 — Nucleotide sequences and location of the C. trachomatis-ompA and N. gonorrhoeae-orf1 genes used to designed the mLAMP primers. [file DataSheet_1.docx]

**Supplementary Materials**

**Visual and rapid identification of *Chlamydia trachomatis* and *Neisseria gonorrhoeae* using multiplex loop-mediated isothermal amplification and a gold nanoparticle-based lateral flow biosensor**

Xu Chen^1, 2Δ*^, Qingxue Zhou^3Δ^, Wei Yuan^4^, Yuanfang Shi^1^, Shilei Dong^5*^, Xinhua Luo^6*^

^1^The Second Clinical College, Guizhou University of Traditional Chinese Medicine, Guiyang, Guizhou, 550003, People's Republic of China

^2^Clinical Medical Laboratory of the Second Affiliated Hospital, Guizhou University of Traditional Chinese Medicine, Guiyang, Guizhou, 550003, People's Republic of China

^3^Clinical Laboratory, Hangzhou Women's Hospital, Hangzhou, Zhejiang 310008, People's Republic of China

^4^Department of Quality Control, Guizhou Provincial Center for Clinical Laboratory, Guiyang, Guizhou, 550002, People's Republic of China

^5^Department of Clinical Laboratory, Zhejiang Hospital, Hangzhou, Zhejiang, 310013, People's Republic of China

^6^Department of infectious disease, Guizhou Provincial People's Hospital, Guiyang, Guizhou, 550002, People's Republic of China

^Δ^Xu Chen and Qingxue Zhou contributed equally to this article.

^*^Corresponding author:

Xinhua Luo, E-mail: luoxh09@163.com

Shilei Dong, E-mail: dsl166@126.com

Xu Chen, E-mail: xuchen1220@126.com

**Supplementary Materials**

**
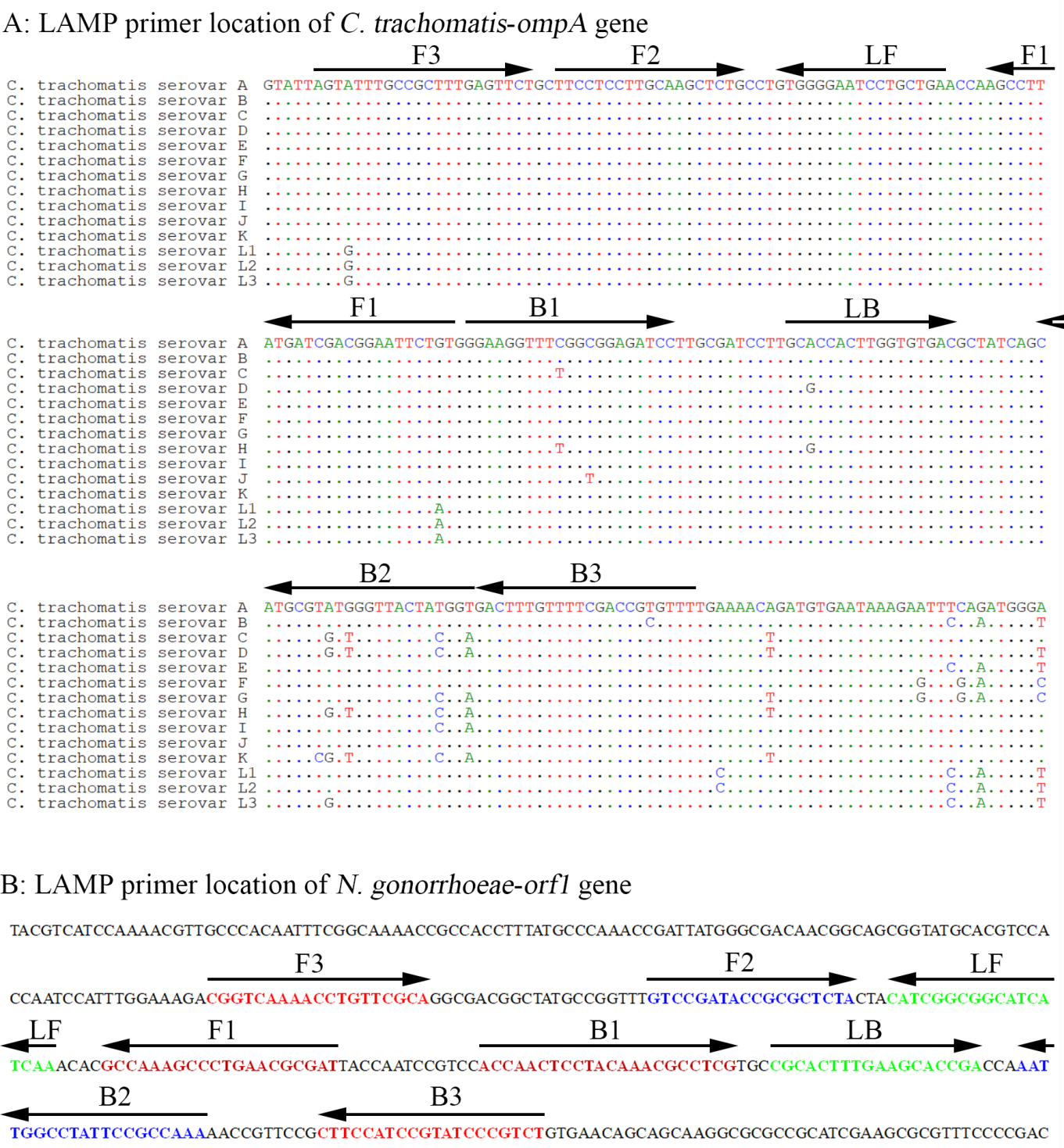
**

**Figure S1. Nucleotide sequences and location of the *C. trachomatis-ompA* and *N. gonorrhoeae*-*orf1* genes used to designed the mLAMP primers**

**(A**) The nucleotide sequences of the *ompA* gene from 14 *C. trachomatis* serological variants (serovar A, B, C, D, E, F, G, H, I, J, K, L1, L2, L3) were aligned with MEGA-X software, and the LAMP primer sequences are marked with arrows. **(B)** The nucleotide sequences and location of *N. gonorrhoeae-*LAMP primers. The right and left arrows showed sense and complementary sequences, respectively.

**Table S1** Comparison of mLAMP-AuNPs-LFB and qPCR methods for assessment of *C. trachomatis & N. gonorrhoeae* in clinical samples

| **Sample No.** | **qPCR^e^ results（copies）** | | **mLAMP-AuNPs-LFB results** | |
| --- | --- | --- | --- | --- |
|  | *C. trachomatis* | *N. gonorrhoeae* | *C. trachomatis* | *N. gonorrhoeae* |
| Test 1 | 4.98×10^5^ | **—** | + | **—** |
| Test 2 | 6.55×10^5^ | **—** | + | **—** |
| Test 3 | 9.32×10^5^ | **—** | + | **—** |
| Test 4 | 1.53×10^5^ | **—** | + | **—** |
| Test 5 | 5.86×10^4^ | **—** | + | **—** |
| Test 6 | 5.06×10^3^ | **—** | + | **—** |
| Test 7 | 776 | **—** | **+** | **—** |
| Test 8 | 2.82×10^6^ | **—** | + | **—** |
| Test 9 | 4.44×10^5^ | **—** | + | **—** |
| Test 10 | 1.69×10^4^ | **—** | + | **—** |
| Test 11 | 1.74×10^5^ | **—** | + | **—** |
| Test 12 | 2.30×10^4^ | **—** | **+** | **—** |
| Test 13 | 4.75×10^5^ | **—** | + | **—** |
| Test 14 | 552 | **—** | + | **—** |
| Test 15 | 1.87×10^5^ | **—** | + | **—** |
| Test 16 | 1.04×10^5^ | **—** | + | **—** |
| Test 17 | 5.82×10^3^ | **—** | + | **—** |
| Test 18 | 1.89×10^5^ | **—** | + | **—** |
| Test 19 | 1.62×10^5^ | **—** | + | **—** |
| Test 20 | 2.45×10^3^ | **—** | + | **—** |
| Test 21 | 1.14×10^5^ | **—** | + | **—** |
| Test 22 | 9.36×10^4^ | **—** | + | **—** |
| **Test 23^a^** | **— (~300)** | **—** | **+** | **—** |
| Test 24 | 3.62×10^4^ | **—** | + | **—** |
| Test 25 | 8.85×10^4^ | **—** | + | **—** |
| Test 26 | 1.39×10^5^ | **—** | + | **—** |
| Test 27 | 3.47×10^4^ | **—** | + | **—** |
| Test 28 | 4.92×10^6^ | **—** | + | **—** |
| Test 29 | 6.72×10^4^ | **—** | + | **—** |
| Test 30 | 1.21×10^5^ | **—** | + | **—** |
| Test 31 | 1.66×10^3^ | **—** | + | **—** |
| Test 32 | 1.34×10^4^ | **—** | + | **—** |
| Test 33 | 7.13×10^5^ | **—** | + | **—** |
| Test 34 | 1.24×10^6^ | **—** | **+** | **—** |
| Test 35 | 2.26×10^6^ | **—** | + | **—** |
| Test 36 | 1.87×10^4^ | **—** | + | **—** |
| Test 37 | 2.82×10^7^ | **—** | + | **—** |
| **Test 38^b^** | **— (~200)** | **—** | + | **—** |
| Test 39 | 1.12×10^5^ | **—** | + | **—** |
| Test 40 | 1.30×10^5^ | **—** | + | **—** |
| Test 41 | 1.12×10^7^ | **—** | + | **—** |
| Test 42 | 1.27×10^4^ | **—** | + | **—** |
| Test 43 | 1.31×10^5^ | **—** | + | **—** |
| Test 44 | 6.96×10^4^ | **—** | + | **—** |
| Test 45 | 2.18×10^5^ | **—** | **+** | **—** |
| **Test 46^c^** | **— (~200)** | **—** | + | **—** |
| Test 47 | 1.57×10^4^ | **—** | + | **—** |
| Test 48 | 1.48×10^3^ | **—** | + | **—** |
| Test 49 | 1.21×10^5^ | **—** | + | **—** |
| Test 50 | 1.41×10^3^ | **—** | + | **—** |
| Test 51 | 1.05×10^6^ | **—** | + | **—** |
| Test 52 | **—** | 8.30×10^2^ | **—** | + |
| Test 53 | **—** | 6.87×10^7^ | **—** | + |
| Test 54 | **—** | 1.25×10^6^ | **—** | + |
| Test 55 | **—** | 3.69×10^7^ | **—** | + |
| Test 56 | **—** | 2.76×10^5^ | **—** | **+** |
| Test 57 | **—** | 3.37×10^5^ | **—** | + |
| Test 58 | **—** | 9.34×10^5^ | **—** | + |
| Test 59 | **—** | 3.36×10^6^ | **—** | + |
| Test 60 | **—** | 6.73×10^6^ | **—** | + |
| Test 61 | **—** | 2.05×10^6^ | **—** | + |
| Test 62 | **—** | 4.2×10^5^ | **—** | + |
| **Test 63^d^** | **—** | **— (~300)** | **—** | **+** |
| Test 64 | **—** | 1.45×10^7^ | **—** | + |
| Test 65 | **—** | 9.37×10^5^ | **—** | + |
| Test 66 | **—** | 5.97×10^6^ | **—** | + |
| Test 67 | **—** | 5.58×10^4^ | **—** | **+** |
| Test 68 | **—** | 4.11×10^4^ | **—** | + |
| Test 69 | **—** | 8.3×10^5^ | **—** | + |
| Test 70 | **—** | 5.13×10^7^ | **—** | + |
| Test 71 | **—** | 8.56×10^4^ | **—** | + |
| Test 72 | **—** | 4.4×10^5^ | **—** | + |
| Test 73 | **—** | 7.61×10^6^ | **—** | + |
| Test 74 | **—** | 8.44×10^6^ | **—** | + |
| Test 75 | **—** | 2.12×10^4^ | **—** | + |
| Test 76 | **—** | 1.08×10^3^ | **—** | + |
| Test 77 | **—** | 9.18×10^2^ | **—** | + |
| Test 78 | **—** | 9.38×10^5^ | **—** | **+** |
| Test 79 | **—** | 4.46×10^5^ | **—** | + |
| Test 80 | **—** | 7.02×10^4^ | **—** | + |
| Test 81 | **—** | 3.85×10^6^ | **—** | + |
| Test 82 | 4.41×10^4^ | 4.63×10^6^ | + | + |
| Test 83 | 9.42×10^2^ | 6.16×10^6^ | + | + |
| Test 84 | 2.03×10^5^ | 4.67×10^4^ | + | + |
| Test 85 | 5.00×10^5^ | 1.40×10^5^ | + | + |
| Test 86 | 1.21×10^6^ | 7.35×10^4^ | + | + |
| Test 87 | 1.44×10^4^ | 2.65×10^6^ | + | + |
| Test 88-146 | **—** | **—** | **—** | **—** |

Notice: ^a^Test 23, ^b^Test 38, ^c^Test 46, ^d^Test 63: the four samples were amplified with PCR (More than one PCR, the first PCR product was used as the template for the next reaction). And then, the final PCR productions were tested through DNA sequencing. The results were consistent with mLAMP-AuNPs-LFB and presented positive outcomes.

^e^qPCR, the qPCR diagnosis was carried out using commercial real-time TaqMan PCR Kit (DaAn Gene Co., Ltd. China). The concentrations of *C. trachomatis* or *N. gonorrhoeae* less than 500 copies will be regarded as negative outcome according to the manufacturer’s instructions.

+, Positive; —, Negative
